# Pre-extensively drug-resistant tuberculosis spondylodiscitis in an immunocompetent patient: a case report

**DOI:** 10.11604/pamj.2020.36.165.21689

**Published:** 2020-07-08

**Authors:** Safaa Fellous, Hanan Rkain, Latifa Tahiri, Amina Bouraqadi, Ittimad Nassar, Fadoua Allali

**Affiliations:** 1 Department of Rheumatology B, El Ayachi Hospital, Ibn Sina University Hospital, Mohammed V University, Rabat, Morocco,; 2 Tuberculosis and Respiratory Disease Diagnostic Center, Bab khmiss, Salé, Morocco,; 3 Central Radiology Department, Ibn Sina University Hospital, Mohammed V University, Rabat, Morocco

**Keywords:** Tuberculosis, spondylodiscitis, drug-resistance, second-line treatment

## Abstract

Pre-extensively drug resistant tuberculosis (pre-XDR-TB) has been an area of growing concern, and posing a threat to global efforts of TB control. We report a case of PreXDR-TB spondylodiscitis with resistance to a Fluoroquinolone, in an immunocompetent patient under antibacillary treatment for pleural tuberculosis, managed with drug sensitivity-based second-line antituberculous drug regimen. Our case shows the challenges of the diagnostic and management of Drug-resistant TB spondylodiscitis.

## Introduction

Tuberculosis (TB) is a communicable disease, one of the top 10 causes of death worldwide. It is a major public-health problem in developing countries, and there are some new challenges due to the emergence of HIV co-infection and drug-resistant tuberculosis [1]. Diagnosis of drug resistant spinal tuberculosis is often delayed resulting in development of spinal deformity and neurological complications [2]. Multidrug-resistant TB (MDR-TB) is TB that is resistant to both rifampicin and Isoniazid. Extensively drug-resistant TB (XDR-TB) is defined as MDR-TB plus a resistance to at least one of the Fluoroquinolones (FQ) and one of the injectable agents used in MDR-TB treatment regimens [1]. PreXDR-TB is defined as MDR-TB with resistance to either an FQ or a second line injectable (SLI), but not both [3]. The burden of pre-XDR-TB is considerable and represents a threat to MDR-TB control [4, 5]. The World Health Organization (WHO) estimated 484 000 incident cases of MDR/RR-TB (MDR or rifampicin-resistant TB) in 2018 [1]. Among MDR/RR-TB patients notified in 2018, 59% were tested for resistance to both Fluoroquinolones and second-line injectable agents, the proportion of MDR/RR-TB cases with resistance to any fluoroquinolone was 20.8% worldwide and the average proportion of MDR-TB cases with XDR-TB was 6.2% [1]. In Morocco, there were 36 000 new cases of tuberculosis in 2018; among these cases, there were 530 new cases of TB-MR/RR, among these 530 patients, 139 were tested for second-line antibacillary drugs [1].

## Patient and observation

A 44-year-old man, chronic smoker, was admitted to the hospital for acute inflammatory lumbar pain which appeared 3 months after the initiation of antibacillary treatment for pleural tuberculosis. Patient was treated with a standard antituberculosis treatment, consisting of Isoniazid (H), Rifampicin (R), Pyrazinamide (Z), and Ethambutol (E) (2 months of RHZE/4 months of RH). The diagnosis of pleural tuberculosis was based on clinical data (weight loss, night sweats, dyspnea) and radiological findings (left pleurisy of average abundance). A pleural biopsy showed epithelioid granulomatous inflammatory process without caseous necrosis, Ziehl- Neelsen stain and culture for mycobacteria were negative. On admission, the clinical examination revealed an afebrile man with a weight of 46kg and BMI of 17.6 kg/m^2^. He had a marked spinal tenderness of the dorso-lumbar spine and important limitation of spinal motion. Our patient did not have respiratory symptoms and his neurologic examination was normal.

**Figure 1:**
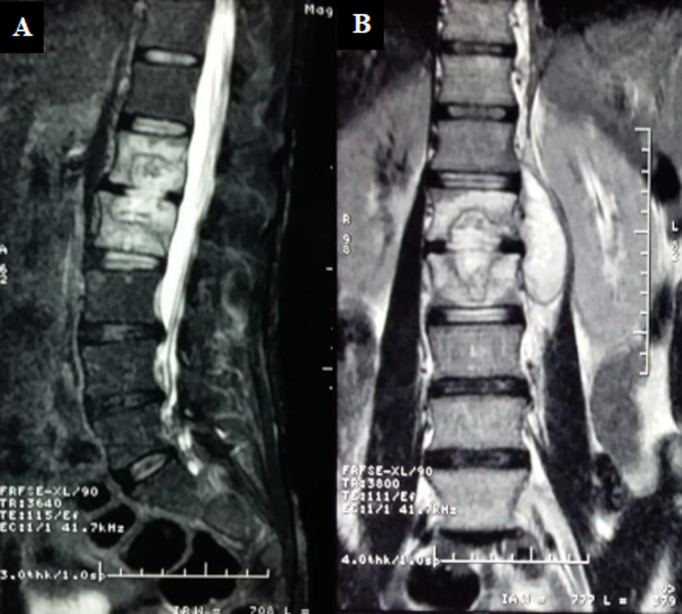
sequential T2-weighted magnetic resonance images of the lumbar spine; A) sagittal; B) coronal views, showing spondylitis L1L2 with collections of the vertebral bodies and in the left psoas

The laboratory evaluation showed a hemoglobin of 12.4 g/dl and a total leucocyte count of 6400/mm^3^with 72% polymorphonuclear cells, 20% lymphocytes. Erythrocyte sedimentation rate (1^st^h) and CRP were respectively 80 mm and 27 mg/l. Sputum acid-fast bacilli direct smear as well as mycobacterium culture were negative. HIV test was also negative. Investigations including an x-ray of lumbar showed osteocondensation concerning the lower shelf of L1 and upper shelf of L2, Chest x-ray showed a small pleural effusion on the left. A dorso-lumbar Magnetic resonance imaging (MRI) showed features suggestive of Ttuberculous (TB) spondylitis interesting lower shelf of L1 and upper shelf of L2, respect for the height of the vertebral bodies with discrete epiduritis. MRI also showed a collection of vertebral bodies L1L2, which communicates with another one abscessed in the left psoas (Figure 1). Computerized tomography (CT) - guided evacuation of a psoas abscess was performed, which revealed purulent material staining negative on Gram stain and Ziehl-Neelsen stain. The cultures of the abscess material showed no growth.

**Figure 2:**
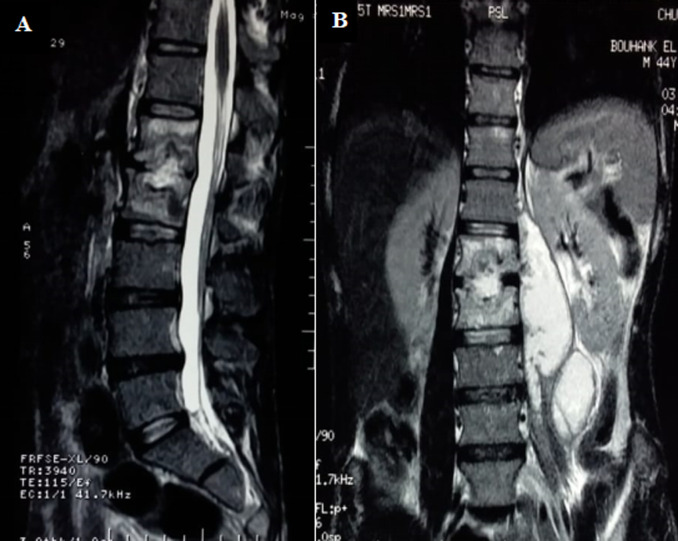
sequential T2-weighted magnetic resonance images of the lumbar spine; A) sagittal; B) coronal views, showing spondylodiscitis L1L2 with a significantly increased size of the left psoas abscess

**Figure 3:**
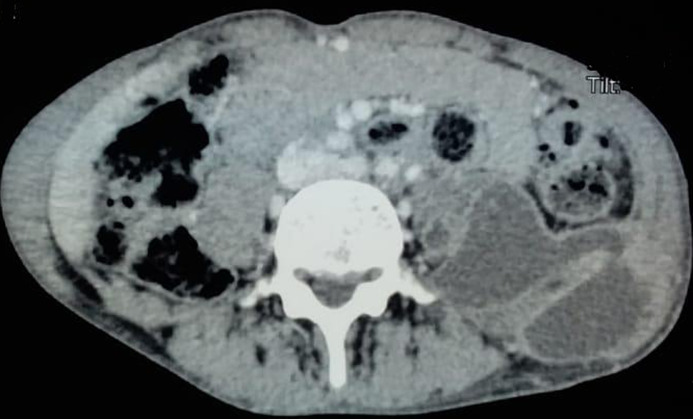
abdomino-pelvic CT showing collections in the left iliac psoas muscle and the left gluteal and the trochanteric region

In view of the high prevalence rate of TB, antecedent of pleural tuberculosis and MRI appearance, the diagnosis of Pott´s disease was retained and the duration of treatment was prolonged to 9 months and the patient was placed in a spinal brace for stabilization of the vertebral column. Despite good adherence to treatment, low back pain worsened. 6 months after the diagnosis of tuberculosis spondylitis, the patient developed purulent collections in the soft tissues in the gluteal and left trochanteric region. A dorso-lombar MRI showed spondylodiscitis L1L2 with a significantly increased size of the left psoas abscess (Figure 2). An abdomino-pelvic CT showed collections in left iliac psoas muscle and the left gluteal and trochanteric region (Figure 3). GeneXpert® MTB/RIF of the vertebral biopsy aspiration product CT-guided revealed a polymerase chain reaction (PCR) for mycobacterium tuberculosis positive, with resistance to Rifampicin. Hain® test of first-line anti-tubercular treatment (ATT) showed resistance to Rifampicin and high-level resistance to Isoniazid. Moreover, Hain® test of second-line ATT (FQ and SLI) showed a high-level resistance to Ofloxacin and sensibility to Kanamycin and Amikacin.

**Figure 4:**
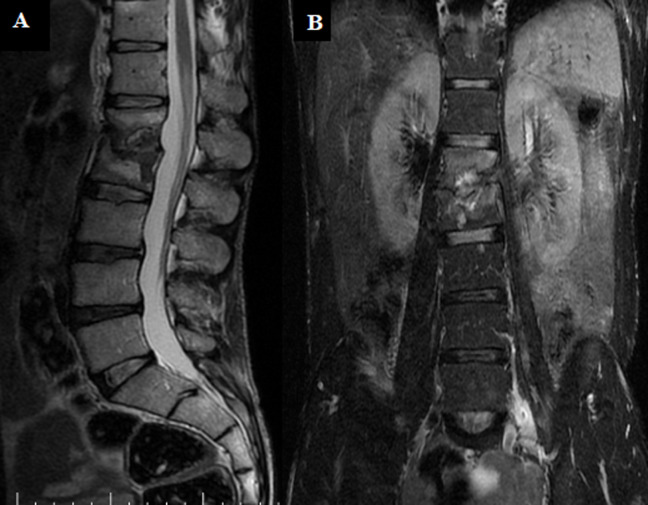
sequential T2-weighted magnetic resonance images of the lumbar spine; A) sagittal; B) coronal views, showing vertebral fractures L1-L2 and a small collection of the left psoas which is atrophied

Our patient was transferred to MDR-tuberculosis Unit. The superficial collections in the left gluteal and the trochanteric region have been drained under ultrasound. Following. An observed second-line treatment was initiated using amikacin, clofazimine, prothionamide, linezolid, delamanide, pyrazinamide et ethambutol, with monitoring for adverse events. At 6 months, injectable Amikacin and Delamanide were stopped, while other oral drugs were continued. Second-line ATT was given for a total duration of 20 months. The evolution under second-line treatment was marked by an improvement in an inflammatory lumbar pain, gain in weight and a disappearance of soft tissue collections. CRP and VS were normalized. Imaging monitoring performed at 12 months of second line treatment, showed no residual collections in thoraco-abdomino-pelvic CT, MRI found a vertebral fracture L1-L2 and quasidisappearance of psoas collection, only a small collection measuring less than 1cm was found without enhancement after gadolinium injection (Figure 4). Our patient received 20 months of treatment that it was tolerated, apart from the paresthesias of the 4 limbs which was managed by vitamin therapy, with stabilization of the vertebral lesions without the appearance of new collections. He was declared cured and he is following a rehabilitation program with stretching and strengthening of the spinal muscles.

## Discussion

Pre-XDR TB has become an area of growing concern, and is posing a threat to global efforts of TB control [4-6]. Drug resistance (DR) is rarely innate, it´s usually the result of inappropriate drug therapy and non-adherence to the treatment. Genetic predisposition and coinfection with HIV positive contribute to the development of drug resistance [2]. The diagnosis of DR TB spondylodiscitis is often delayed, with a delay that varies between 6 months and 2 years [7-9]. Drug-resistant tuberculous spondylodiscitis should be suspected in patients showing a poor clinical and radiological response or appearance of a fresh lesion of osteoarticular TB or deterioration of spinal deformity while on ATT for a minimum period of 5 months [10]. In our patient, drug resistant was suspected in the aggravation of lumbar pain, appearance of collections in the soft tissues and increase of volume of abscess of psoas showed by MRI. Spinal TB is a deep-seated paucibacillary lesion. Detection DR-TB requires bacteriological confirmation of TB and testing for drug resistance using rapid molecular tests, culture methods or sequencing technologies [2]. In our patient, the culture of mycobacterium tuberculosis was negative, the diagnostic of Pre XDR-TB was made using GeneXpert MTB/RIF that showed a resistance to rifampicine, completed by sequencing technology with Hain® test of first and second-line ATT (FQ, SLI). Those genetic tests showed resistance to Rifampicine, Isoniazid and Ofloxacin.

Treatment for MDR-TB is complex and it requires considerable skills and resources. WHO has developed and issued recommendations on the treatment and care of patients with drug resistant-TB with an up to date in 2019 [11]. Treatment of MDR-TB is long with a total duration of up to 20 months, with an intensive phase and a maintenance phase, the treatment is based on second-line drugs with a well codified protocol. Patients with resistance to additional anti tubercular drugs should receive individualized ATT following their drug sensitivity testing results [11]. Our patient had a resistance to Rifampicin, Isoniazid and ofloxacin, he was treated by an individual protocol, using Amikacin, Clofazimine, Prothionamide, Linezolid, Delamanide, Pyrazinamide and Ethambutol for a total period of 20 months. The evolution of multidrug-resistant tuberculosis treated is less favorable than that of susceptible tuberculosis and depends largely on the duration of treatment and the quality of monitoring and care. The latest treatment outcome data for people with MDR/RR-TB shows a global treatment success rate of 56%, WHO reported a mortality prevalence of 15% in this population [1]. In Morocco, among the patients who started second-line treatment in 2016, the proportion of MDR/RR-TB patients who successfully completed treatment is 55% [1]. In our patient, the evolution under treatment was favorable and he was declared cured.

## Conclusion

The prognosis of drug resistant tuberculosis is serious, the burden of pre-XDR-TB is considerable and represents a threat to MDR-TB control. Our patient illustrates the problem of late diagnosis and the difficulty of the therapeutic management. Pre-XDR-TB should be suspected if there is a clinical and/or radiological progression of TB in spite of an appropriate antitubercular treatment. Effective treatment of pre-extensively drug-resistant TB requires a complex and prolonged drug sensibility-based anti-tubercular treatment.
